# Primary Human *Streptococcus canis* Bloodstream Infection Linked to Cat Contact: A Case Report and Review of Literature

**DOI:** 10.1155/crdi/3375049

**Published:** 2026-08-10

**Authors:** Abdulaziz M. Alshalan, Abdullah F. Alotaibi, Monirah A. Alamash, Sarah S. Alangari, Maha M. Almohizea, Mazin Barry

**Affiliations:** ^1^ Department of Internal Medicine, King Saud University Medical City, Riyadh, Saudi Arabia, ksu.edu.sa; ^2^ Division of Gastroenterology, King Saud University Medical City, Riyadh, Saudi Arabia, ksu.edu.sa; ^3^ King Fahad Cardiac Center, King Saud University Medical City, Riyadh, Saudi Arabia, ksu.edu.sa; ^4^ Infectious Diseases Unit, College of Medicine, King Saud University, Riyadh, Saudi Arabia, ksu.edu.sa; ^5^ Department of Pathology, College of Medicine, King Saud University, Riyadh, Saudi Arabia, ksu.edu.sa; ^6^ Division of Infectious Diseases, Faculty of Medicine, University of Ottawa, Ottawa, Canada, uottawa.ca

**Keywords:** bacteremia, bloodstream infection, cat contact, *Streptococcus canis*, zoonosis

## Abstract

**Introduction:**

*Streptococcus canis* is a rare zoonotic pathogen reported to cause human disease, including skin and soft tissue infections, bloodstream infections, and infective endocarditis. The majority of cases have been linked to contact with dogs, with a paucity of cases related to cats. It is generally susceptible to penicillin and vancomycin but exhibits a high rate of resistance to erythromycin and an inducible resistance to clindamycin.

**Case Presentation:**

A man in his sixties presented with fever, chills, and a diabetic foot ulcer that had been exposed to a cat. He was found to have *S. canis* primary bloodstream infection, which showed a typical susceptibility to beta‐lactams and vancomycin, and resistance to clindamycin and macrolides. He was treated with ceftriaxone and had no recurrence at the 3‐month follow‐up.

**Conclusion:**

*S. canis* related to cat exposure is an extremely rare cause of human infection, in such cases, a beta‐lactam antimicrobial should be initiated. In individuals with an allergy to beta‐lactams, vancomycin should be used with avoidance of macrolides and clindamycin as empirical therapy due to the high resistance rate to these antimicrobials among *S. canis*.

## 1. Introduction

*Streptococcus canis* (*S. canis*) is a Gram‐positive β‐hemolytic, Lancefield group G pyogenic coccus in chains that was first reported in 1937 after being isolated from dead puppies and female dogs’ genital tracts [1]. Other than dogs, the organism has been isolated from a wide variety of mammals, including rabbits, seals, sea lions, badgers, and cats. The differentiation between *S. canis* and *Streptococcus dysgalactiae* (*S. dysgalactiae*) *subsp. equisimilis*, which is a human‐specific pathogen, was challenging as both belong to Lancefield group G *streptococci*; however, several phenotypic and biochemical traits distinguish these two species including hyaluronidase and fibrinolysin activity that is present in *S. dysgalactiae subsp. equisimilis* and absent in *S. canis*; α‐ and β‐galactosidase which are active in *S. canis* and absent in *S. dysgalactiae subsp. equisimilis*, in addition, to 16S rRNA gene sequencing differentiation which is the only way to be identified in case of negative culture [2–5].

Humans have also been reported to be infected with *S. canis* with a wide variety of infections, including skin and soft tissue infections, infective endocarditis, prosthetic joint infections, and bloodstream infections. Most reported infections were linked to dogs as this organism is known to colonize their oropharynx [5]. Although cats can also be colonized with *S. canis*, there is a paucity of cases linked to them [6]. We report a rare case of *S. canis* primary bloodstream infection linked to a cat.

## 2. Case Presentation

A man in his sixties presented to our institution’s Emergency Department in October 2024 with one day history of fever, chills, and rigors. His past medical history includes hypertension, diabetes mellitus for 40 years with micro‐ and macrovascular disease, chronic kidney disease, coronary artery disease status postcoronary artery bypass grafting, ischemic cardiomyopathy, peripheral artery disease‐status post–right aortofemoral bypass surgery in 2006, and a 50‐pack year of tobacco smoking. Six months prior to this presentation, he had a left diabetic foot infection involving his heel. He underwent incision and drainage of an abscess with debridement and was treated with oral amoxicillin/clavulanic acid for 6 weeks; his heel continued to heal. He continued to undergo daily dressing and wound care at home. A few days prior to his illness, his family adopted a street cat that had regular direct contact with his exposed wound. However, no scratches or direct licking were reported. He had no other animal contacts, and he never had any contact with any dogs.

**FIGURE 1:**
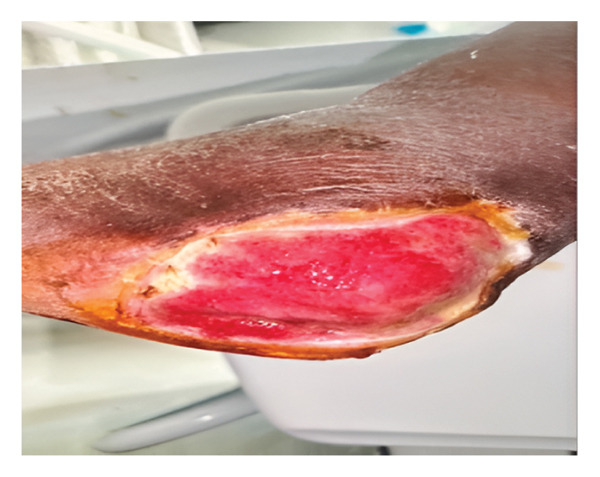
Well‐defined clean‐based wound with healthy granulation tissue.

On physical examination, he looked unwell, vital signs: temperature 39.5°C, heart rate 131 beats/minute, blood pressure 160/70 mmHg, respiratory rate 20 breaths/minute, oxygen saturation 86% on room air, and 94% on 3 L via nasal cannula. The left heel (Figure 1) showed a lateral ulcer that was clean and dry, with good granulation tissue, no discharge, with clear borders, and without any surrounding erythema or tenderness. The site of the right aortofemoral bypass was clean, with no discharge from the surgical wound, without any tenderness, and its pulsation was palpable. The chest exam revealed bilateral diffuse crackles, with reduced air sounds. A cardiac examination revealed a Grade 3/6 holosystolic murmur at the left lower sternal border and a raised jugular venous pressure. The abdominal exam was soft with no palpable hepatosplenomegaly.

His significant lab investigations include a white blood cell count (WBC) of 23.15 × 10^9^/L (normal 4–11 × 10^9^/L) with neutrophils of 22.5 × 10^9^/L (normal 2–7.5 × 10^9^/L), erythrocyte sedimentation rate (ESR) 85 mm/h (normal 01–17 mm/h), C‐reactive protein (CRP) 132.52 mg/L (normal less than 10 mg/L), creatinine 602 μmol/L—within patient’s baseline— and glucose 22.05 mmol/L (normal 4.56–6.38 mmol/L). A chest X‐ray showed bilateral congestion with signs of pulmonary edema. He was admitted to the hospital, two sets of blood cultures were drawn, he was given diuresis and insulin, and was started empirically on intravenous (IV) ceftriaxone with a dose of 2 gm once daily.

**FIGURE 2:**
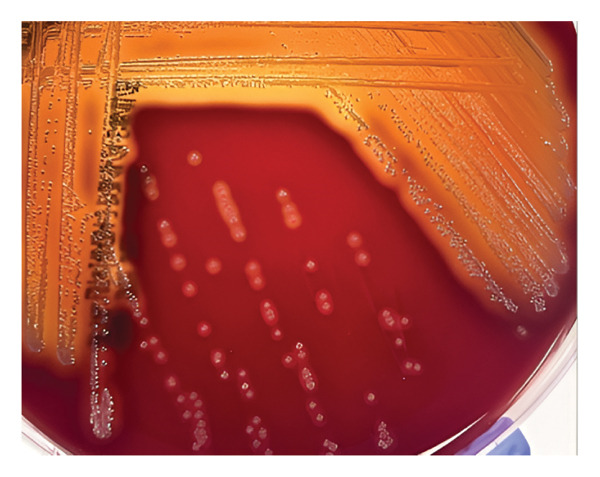
Small‐medium sized, grayish‐white, smooth, glossy, translucent beta hemolytic colonies of *S. canis* growing on sheep blood agar (SBA) after 24 h incubation.

Within 24 h, the aerobic blood culture bottle from one set flagged positive by the Blood Culture Continuous Monitoring System (BACT/ALERT VIRTUO | BioMérieux). Gram staining was performed, showing Gram‐positive cocci arranged in chains. It was then cultured on sheep blood agar, chocolate agar, and MacConkey agar plates (Saudi Prepared Media Company, Riyadh, Saudi Arabia). The sheep blood agar and chocolate agar plates were incubated at 35 ± 1°C with 5% CO_2_, while the MacConkey plates were incubated at 35 ± 1°C in ambient air. After the 24‐h incubation period, growth was detected on the sheep blood agar and chocolate agar plates. Particularly, the growth on the sheep blood agar demonstrated small, grayish‐white, glossy, and translucent beta hemolytic colonies (Figure 2). The microorganism was identified using Matrix‐assisted laser desorption ionization‐time of fight mass spectrometry (MALDI‐TOF MS) as *S. canis* with 99.9% confidence level via the VITEK 2 system GP card (BioMérieux, Marcy‐L’Etoile, France).

Susceptibility results to antibiotics were also provided by the same VITEK 2 system using the AST‐ST03 panel. The organism showed susceptibility to penicillin, ceftriaxone, vancomycin, and sulfamethoxazole. However, it exhibited an inducible clindamycin resistance and resistance to erythromycin.

The patient was afebrile 1 day after admission, and a repeated blood culture did not grow any organism after 5 days of incubation. A transesophageal echocardiogram showed an ejection fraction of 30%–35% with mild sclerotic valvular changes, moderate functional mitral regurgitation, and moderate tricuspid regurgitation; no vegetations were detected. He continued to be treated with IV ceftriaxone 2 gm once daily for a planned 2‐week course. He was discharged in good condition 7 days into treatment. Upon discharge, his repeated CRP was 16 mg/L, and WBC was 6.2 × 10^9^/L. At one‐ and 3‐month follow‐up in the clinic, he was doing well, with no recurrence of fever.

## 3. Discussion and Conclusion

*S. canis* is a rare and underreported organism that colonizes mammals, especially dogs and reportedly cats [2]. Veterinary medicine reports higher colonization of *S. canis* in diseased cats that can be as high as 20.8% compared to healthy cats [5], in addition, the infectiousness can be increased if the colonized pet had an active infection when in direct contact with a susceptible human host including those above 65‐year‐old with diabetes mellitus.

Beyond bacteremia, majority of the reported cases are related to skin and soft tissue infections [4, 7–9]; however, osteomyelitis, sacroiliitis, and myositis have also been described as a metastatic complication of *S. canis* bacteremia in a patient with regular household dog contact but no bite or scratch [10]. Prosthetic joint infection is a further recognized manifestation, illustrated by cases identified only after next‐generation sequencing or extended culture, again typically linked to a dog bite or lick over a preexisting wound [11, 12]. Infective endocarditis, as summarized in Table 1, is a well‐documented and potentially severe presentation, most often affecting native or prosthetic aortic and mitral valves in older adults with underlying cardiac disease [6, 15, 17, 18].

**TABLE 1:** Reported cases with bloodstream infection and type of animal contact.

Case report	Age, gender, and comorbidities	Animal contact	Antibiotics susceptibility/resistance	Treatment	Presentation/outcome
Bert and Lambert‐Zechovsky [13]	77‐year‐old man with hypertension and continuous arrhythmia	Owning a dog	S: Benzylpenicillin, amoxicillin, cefalotin, erythromycin, clindamycin, pristinamycin, rifampin, and vancomycin and R: tetracycline	Intravenous (IV) amoxicillin (6 g/day) × 2 weeks followed by 10 days of Oral amoxicillin (3 g/day)	Recovered

Tekeda et al. (2001) [14]	75‐year‐old woman with mitral valve replacement	Dog bite of an exposed wound	S: Ampicillin and netilmicin	IV ampicillin (8 g, 4 times per day) and netilmicin (30 mg, once a day) × 28 days	Cellulitis Recurred within 3 weeks then Recovered

Lam et al. [7]	75‐year‐old man with chronic osteomyelitis secondary to pressure ulcer	Owning a dog	S: Erythromycin and penicillin	IV clindamycin and ceftazidime	Recovered

Takamura et al. [15]	82‐year‐old woman with aortic valve bioprosthesis	Not mentioned	Not mentioned	IV penicillin G and gentamicin, valve rereplacement	Prosthetic aortic valve endocarditis Recovered

Ohtaki et al. [16]	91‐year‐old woman with hypertension and femoral neck fracture	Owning a dog without a bite	S: Penicillin G, ampicillin, cefotaxime, ceftriaxone, cefepime, imipenem, erythromycin, minocycline, sulfamethoxazole/trimethoprim, and levofloxacin	IV ceftriaxone 2 g/day × 2 weeks	Recovered

Amsallem et al. [17]	65‐year‐old woman with uterine carcinoma	Dog contact without bite	S: penicillin with low‐level resistance to gentamicin	IV amoxicillin 200 mg/kg/day × 6 weeks, IV gentamicin 3 mg/kg/day × 2 weeks, Valve replacement	Native (mitral) valve infective endocarditis Recovered

Lacave et al. [18]	71‐year‐old man with dilated cardiomyopathy due to chronic ethanol abuse, hepatic cirrhosis, and diabetes	Dog licking of an exposed wound	S: Amoxicillin low‐level R: gentamicin	IV amoxicillin 200 mg/kg/day (6 doses/day) × 2 weeks followed by 3 weeks of oral amoxicillin	Native (aortic) valve infective endocarditis Recovered

Cheong and Lim [8]	57‐year‐old man with hypertension and diabetes	Regular close contact with dog	S: Cefotaxime, ceftriaxone, clindamycin, erythromycin, and penicillin. R: Tetracycline	IV sulbactam/ampicillin combination and oral Clindamycin, then upgraded to IV meropenem few days deescalated to IV ceftriaxone 2 g/day all total of 11 day followed by oral sulbactam/ampicillin × 7 days	Left foot abscess recovered

Taniyama et al. [19]	71‐year‐old man with hypertension, liver cirrhosis, splenectomy	Dog bite	S: Penicillin, cephalosporin, tetracyclines (TCs), glycopeptides, lincomycins, sulfamethoxazole/trimethoprim, chlor‐ amphenicol, and fluoroquinolones. Erythromycin Intermediate S: azithromycin	IV ampicillin (12 g per day, administered as six 2 g doses) and clindamycin (1800 mg per day, administered as three 600 mg doses) × 2 weeks	Left foot abscess recovered

Zaidi and Eranki [9]	62‐year‐old man with renal transplant, congestive heart failure, pituitary adenoma, hypothyroidism, diabetes, obstructive sleep apnea	Dog licking of an exposed wound	S: Penicillin	IV ceftriaxone 2 g/day × 14 days	Recovered

Afzal et al. [20]	85‐year‐old female with chronic lymphedema, chronic venous stasis, daily alcohol use.	Dog licking of an exposed wound	Not mentioned	IV piperacillin/tazobactam upgraded to IV cefepime 2 g/day then switched to ceftriaxone × 6 weeks then oral amoxicillin until repeated negative Transthoracic echocardiogram	Right atrium infective endocarditis Recovered

Wang et al. [6]	74‐year‐old male with Type 2 diabetes mellitus, agent orange exposure, prior calcaneal osteomyelitis, CKD3a, CHF, and moderate aortic stenosis	Cat licking to an exposed wound	S: Ampicillin, cefotaxime	IV Ceftriaxone 2 g/day × 6 weeks	Native (aortic) valve infective endocarditis Recovered

Tsai et al. [21]	64‐year‐old man with hypertension, coronary artery disease, nasopharyngeal carcinoma in remission, hyperlipidemia, gout and chronic hepatitis B.	Lives with a dog	S: Ceftriaxone	IV Ceftriaxone 2 g every 12 h × 4 weeks	Recovered

Van Tol et al. [10]	2‐year‐old man; previously healthy	Lives with a dog	Pan‐susceptible	IV ampicillin × 2 weeks then IV penicillin G × 6 weeks	Cellulitis, osteomyelitis, sacroiliitis, myositis Recovered

This report	A man in his early 60s with hypertension, diabetes, CKD4 and peripheral vascular disease.	Regular close contact with cat	S: Penicillin, ceftriaxone, vancomycin and sulfamethoxazole. R: erythromycin; inducible R: clindamycin	IV ceftriaxone 2 g/day × 14 days	Recovered

*Note:* Summary of Table 1 (*n* = 15 cases including the present report): Twelve cases (80%) were associated with dog contact or ownership, two (13.3%) with cat contact, and one report did not mention animal contact. Where susceptibility data were reported, isolates were uniformly susceptible to penicillin, and to ceftriaxone in all cases where it was tested. Clinical presentations included uncomplicated bacteremia (*n* = 8), infective endocarditis (*n* = 5, native or prosthetic valve), and bacteremia with metastatic musculoskeletal/soft tissue involvement, i.e., osteomyelitis, sacroiliitis, myositis, or abscess (*n* = 2). All 15 patients survived to discharge (100% recovery); one patient experienced clinical recurrence, which resolved with retreatment.

The presented case developed a primary bloodstream infection a few days after close, regular direct contact with a cat to his exposed wound without direct licking or scratching. We hypothesize that this open wound was the source of entry of the bacteria that the newly adopted street cat was most likely colonized with. A recent case of *S. canis* native valve infective endocarditis has been linked to a cat after licking an exposed wound [6]. In contrast to previous reported cases of *S. canis* primary bloodstream infections, which were linked to dogs with no direct contact in most (Table 1).

Of the fifteen bloodstream infection cases summarized in Table 1, twelve were associated with dog contact or ownership and only two including our case have been linked to cat contact; in one case, animal contact was not documented. Where antimicrobial susceptibility was reported, isolates were consistently susceptible to penicillin and, where tested, to ceftriaxone, consistent with the pattern described in the wider literature. All fifteen patients survived to hospital discharge with antimicrobial therapy, with recurrence in only one case, which subsequently resolved with retreatment.

The prevalence of *S. canis* infection remains low, and the virulence mechanisms are not well understood; however, it has been shown to have many virulence factors such as Neuraminidase B, Streptolysin O, and Streptolysin S. One virulence factor that has been well studied is *S. canis* M‐like (SCM) protein, which contributes to tissue adhesion, invasion, and antiphagocytic effect; however, the clinical significance of SCM in clinical infection in animals has been debated [5, 11].

*S. canis* is susceptible to beta‐lactam antibiotics and have been successfully treated with penicillin, ampicillin, and amoxicillin. It is also susceptible to vancomycin; however, up to 40% of all isolates are resistant to tetracycline, macrolides, and lincosamide resistance. The latter has been observed in strains that carry the *erm* genes [4, 5]. The isolate in the current case showed resistance to erythromycin and an inducible clindamycin resistance. Hence, the empiric use of these agents when *S. canis* infection is suspected should be avoided. Clinicians should suspect *S. canis* bloodstream infection in febrile diabetic patients who have contact with dogs, and similarly to cats [22].

## Author Contributions

Abdulaziz M. Alshalan: corresponding Author, conceptualization, visualization, writing–original draft, and writing–review and editing. Abdullah F. Alotaibi: writing–original draft and writing–review and editing. Sarah S. Alangari, Monirah A. Alamash, and Maha M. Almohizea: writing–original draft. Mazin Barry: conceptualization, visualization, writing–original draft, writing–review and editing, and supervision.

## Funding

The authors received no funding for this work.

## Ethics Statement

This case report was approved by the Institutional Review Board, King Saud University, Riyadh, Saudi Arabia.

Ethical approval number: E‐24‐9354.

## Consent

Written informed consent was obtained from the patient for publication of this case report and accompanying image.

## Conflicts of Interest

The authors declare no conflicts of interest.

## Supporting Information

Additional supporting information can be found online in the Supporting Information section.

## Supporting information


**Supporting Information** This case report was prepared in accordance with the CAse REport (CARE) guidelines for case report reporting [23]. The completed CARE checklist is provided as a supporting information accompanying this submission.

## Data Availability

All data generated or analyzed during this study are included within the published article.
